# An Economic Analysis of the Costs Associated with Pre-Weaning Management Strategies for Dairy Heifers

**DOI:** 10.3390/ani9070471

**Published:** 2019-07-23

**Authors:** Anna Hawkins, Kenneth Burdine, Donna Amaral-Phillips, Joao H.C. Costa

**Affiliations:** 1 Department of Animal & Food Science, University of Kentucky, Lexington, KY 40506, USA; 2 Department of Agricultural Economics, University of Kentucky, Lexington, KY 40506, USA

**Keywords:** calf economics, replacement, ADG, cost per kg

## Abstract

**Simple Summary:**

Rearing of replacement female calves on a dairy farm is of critical importance to maintain herd sizes, improve the genetic quality of the herd, and remain economically sustainable. A 2-year investment period is needed for replacement female heifers to grow before entering the milking herd. The management of replacements over this 2-year period can vary greatly among operations, making it difficult to compare producers’ cost to benchmark. The objective of this project was to develop a model to calculate the cost of rearing a replacement heifer from birth to weaning under different housing, milk source, allotments, and labor and health management decisions to be used as a dairy farm decision support tool. We calculated the cost for management options with general cost values. We found that the average feed cost represented 46% of the total cost while labor, and fixed and variable costs represented 33%, 9%, and 12%, respectively. The total cost increased as milk allotment increased, but cost per Kg of gain decreased. The ranges in total cost within each management scenario often exceed the difference in cost from one scenario to the next. In conclusion, variable costs have the potential to vary among operations, playing a major role in the total cost of rearing replacements from birth to weaning.

**Abstract:**

Dairy calves are raised in various housing and feeding environments on dairy farms around North America. The objective of this study was to develop a simulation model to calculate the cost of raising replacement dairy heifers using different inputs that reflect different management decisions and evaluate their influence on the total cost. In this simulation, 84 calves were modeled between 0–2 months of age to reflect a 1000 heifer herd. The decisions associated with housing, liquid diet source and allowance, labor utilization, and health were calculated. Costs and biological responses were reflective of published surveys, literature, and market conditions. A 10,000-iteration economic simulation was used for each management scenario using @Risk and PrecisionTree add-ons (Palisade Corporation, Ithaca, NY, USA) to account for variation in pre-weaning mortality rate, weaning age, and disease prevalence. As milk allotment increased, total feed cost increased. Feeding calves a higher allowance of milk resulted in a lower cost per kg of gain. Average feed cost percentage of the total cost was 46% (min, max: 33%, 59%) while labor, and fixed and variable cost represented 33% (20%, 45%), 9% (2%, 12%), and 12% (10%, 14%), respectively. Total pre-weaning costs ranged from $258.56 to $582.98 per calf across all management scenarios and milk allotments.

## 1. Introduction

Heifer availability is critical for the dairy operation to maintain a consistent herd size and remain economically sustainable in most cases [1]. Improved fertility and increased use of sexed semen have made replacement heifers more available for dairy operations [2]. Some producers keep all newborn replacement heifers in case more replacements are needed than anticipated, which can create a heavy financial burden for producers when raising excess heifers. Heifer raising expenses are often lumped into broad farm-wide expenses such as feed, labor, and health costs, making it difficult to accurately calculate heifer raising costs [3]. In addition, failing to identify the on-farm cost to raise a replacement heifer can allow for inefficiencies in feed, labor, housing, or health costs to go unnoticed, which accumulate unanticipated replacement female costs.

Previously reported replacement heifer rearing costs are variable and can be explained in part by differences in rearing management systems. For example, the average total cost to raise replacement heifers to wean was found to only increase by $82.88 per heifer from 2000 to 2015 but ranges within each study can exceed $350 per heifer [4,5,6]. Heinrichs [6] found a range in feed cost on 44 farms of $29.06 to $259.17 per calf; total cost per calf ranged $89.00–$442.78 during the pre-weaning period. In a 2014 survey of 2545 heifer calves in the United States, individual housing was the dominant form of housing pre-weaned heifers at 86.6% and 13.4% were managed in group housing, yet 8 different housing types were reported [7]. Little research has examined the cost between housing types, although the University of Wisconsin has conducted surveys of producers in an automatic and conventional housing scenario. The average cost (min, max) of producers utilizing individual housing was $363.69 ($195.06, $530.76) and those with group housing was $401.58 ($138.39, $585.52), a difference in average cost of $37.89 per calf, but with a difference range of over $300 for individual and $400 for group housing [8].

Housing is the first of many decisions a producer makes on how pre-weaned calves will be managed. Utilization of labor and milk source requires additional decisions based on resources and availability. While gaining in popularity, only 1.9% of the calves were fed through an automatic feeder while almost half of the surveyed calves were fed using a bottle or a bucket [9]. On average, one calf requires 7–12 labor hours during the pre-weaning period, or 7–10 mins per day [8]. Unpasteurized whole milk was the most common milk source utilized by producers but close to 50% of those also utilized milk replacer [7]. More recent surveys show a similar trend, with 40.1% of calves fed whole or waste milk, 34.8% fed milk replacer, and 25.1% fed a combination of the two. Calf starter was provided, starting on average at 5 days old, to all calves surveyed [9]. 

Thus, it is important to understand the costs associated with the myriad of rearing systems for dairy calves in the United States. The objective of this paper was to evaluate the economic impact of different calf raising management decisions, especially housing, liquid diet and allowance, and health expenses on the total pre-weaning cost of rearing heifer replacements.

## 2. Materials and Methods

**Figure 1:**
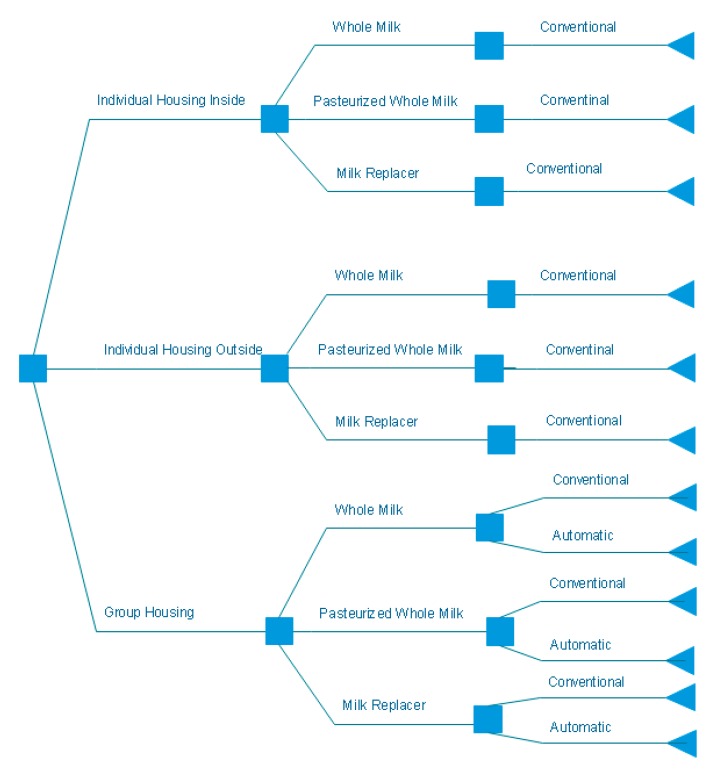
Decision tree of possible management decisions for housing, milk source, and labor for pre-weaned calves.

A cost simulation model was developed at the University of Kentucky Dairy Science program during 2018. This economic model was developed in Excel 2013 (Microsoft, Redmond, WA, USA) utilizing @RISK and PrecisionTree add-ons (Palisade Corporation, Ithaca, NY, USA). The base herd used included 1500 milking cows, 1000 replacement heifers in total and 84 heifer calves in the pre-weaning period, assuming a 30% replacement rate and an average age at first calving of 25 months. Costs were calculated on a per head basis for housing, feed, labor, mortality, and health. All remaining variables are static. Interest was accounted for on infrastructure and mortality as well as the depreciation of assets related to replacement females. Costs associated with herd-wide parameters, such as disease prevalence and mortality rates, were distributed across all remaining calves in the pre-weaning phase. The model required a management decision at 3 points: housing type, milk source, and labor shown in Figure 1. Three main housing types were modeled: individual housing outside (IHO), individual housing inside (IHI) [10], and group housing (GH). Three milk sources were built into the model: whole milk (WM), pasteurized whole milk (PWM), or milk replacer (MR). Four possible liquid feeding plans were modeled: 6, 8, 10, and 12 L of milk per calf per day. Labor was modeled for two categories: conventional, where a person was assigned to feeding and caring for the calves; or automatic, where an automatic calf feeder was utilized in addition to human labor. Totals costs were reported per calf for each management decision, the entire pre-weaning period per calf, and per day per calf. Per day cost was calculated by dividing days of age at weaning by the total cost per calf during the pre-weaning period.

### 2.1. Housing

Housing systems that required a barn (IHI and GH) used values found from Table 1 to determine barn value and monthly payment. Barn cost was derived from the Dairy Calf and Heifer Association Gold Standard recommendation of 3.3 M^2^ per calf, with an additional 15% of space to account for walkways and feed areas. Thus, replacement heifers were assumed to require 3.7 M^2^ per calf. Construction cost [11] varied based on the infrastructure required for each situation, ranging from $10.00 to $15.50 per M^2^. Estimated barn value (BV) was then calculated with Equation (1).

BV = CC × 3.7 M^2^ × number of pre-weaned calves (1)

Barn cost per heifer (BC) was calculated using the payment function in excel with 7% interest, 20 years useful life and BV. BC was included in IHI and GH situations. Calves housed in individual housing outside followed the same payment function. Housing calves year-round in individual housing with an average occupancy time of 2 months ± rest period would allow 5 calves per hutch per year. Days of age at weaning was used as the length of time a heifer was incurring cost during the pre-weaning period. Housing costs also included utility costs, such as water and electricity. Electricity was only factored for housing systems that included a barn (IHI and GH). The bedding was included at a flat evaluation of $11.00 per calf. For pasture scenarios, a cash value price per acre was used as the value of land to try to account for the opportunity cost of a specific acre being used for other purposes. An additional annual maintenance cost of $31.50 per acre was assumed.

### 2.2. Feed

Milk replacer was mixed at a concentration of 0.11 kg per liter of water. A pasteurizer was depreciated over all calves over the 15-year useful life. The model accounted for four possible feeding milk allotments: 6, 8, 10, and 12 L per calf per day. A 2016 survey of producers in the United States showed over half of the farms were feeding calves between 4–6 L per day [7]. Recent studies have shown that increasing milk allotment can increase average daily gain (ADG) pre-weaning, result in larger skeletal measurements at weaning, and decrease vocalizations caused by milk deprivation [12,13]. Milk allotments and starter intakes per calf for this model were reflective of experimental data [14]. In this study, calves were randomly assigned to 6, 8, 10, or 12 L feeding treatments of pasteurized whole milk with *ad libitum* access to calf starter. A step-down weaning program was performed: milk was fed at maximum allotment until weaning at 42 days. Milk allotment was decreased by 50% until day 50, where allotment was decreased daily by 20% until weaned. Calves were assumed to be consuming at least 2.25 kg of calf starter at weaning. An additional 20% was assumed to be fed to account for waste and loss. This additional expense was added to daily calf starter cost. Milk and calf starter costs were calculated on a daily basis for the entire pre-weaning period, from day 0 to 65. ADG was determined using the dry matter intake requirements and resulting gain from NRC, 2001. Calf weight was modeled daily to determine appropriate weaning weights based on dry matter intake from milk replacer or whole milk.

The assumed birth weight was 40 kg for each calf. Assumed average daily gains on each feeding plan (6, 8, 10, and 12 L) are described in Table 2, following the equation presented in NRC, 2001. The weaning weight was calculated by multiplying ADG by 65 days and adding the weight gain to BW. Feed cost was reported for three variables: total cost during the pre-weaning period, daily feed cost, and feed cost per kg of gain. Total feed cost included milk replacer or whole milk expenses and feeding equipment. Daily feed cost was derived from dividing total feed cost by weaning age (65 days). Daily feed cost was then used to calculate feed cost per kg of gain. Daily cost under each feeding plan was divided by ADG to determine the cost per kg of gain. 

### 2.3. Labor

Labor to care for calves and the number of employees working were adapted from published surveys of producers employing individual and group housing (Table 1). Because of the lack of data on group housing without automatic feeder labor time requirements, we assumed the median of an automatic feeder and individually housed heifers (5.5 mins/calf/day). Management labor was calculated separately to represent additional labor required from owners, managers, and/or family. Management followed the trend of 10% of the paid labor, creating the assumption of 0.55 mins/calf/day for group housing without an automatic feeder. Minutes per calf could be input directly or total time per all pre-weaned calves could be used to calculate total labor cost using Equation (2).

((Daily Paid Labor Hours/Number of Calves) × Hourly Paid Labor) + ((Daily Management Labor Hours/Number of Calves) × Hourly Management Pay)(2)

The expenses related to buying and using an automatic calf feeder were included in the labor section. Justified by the change in labor demands, the use of an automatic calf feeder can be viewed as an additional autonomous employee. The cost of the feeder was assumed at $15,000 value, 10 years useful life and $200.00 annual maintenance. These values were assumed based on market prices and a routine maintenance program. Equation (3) represents the calculation of daily feeder cost per calf using the payment (PMT) function in excel.

(−PMT (interest rate, 120, initial value))/number of pre-weaned calves(3)

### 2.4. Mortality and Health

The cost of each calf was calculated daily and, therefore, monthly cost to raise one calf in each management style was determined. All calf mortality events were assumed to occur at the end of the first month of life, accruing the additional monthly cost plus interest. This additional cost is divided over the remaining number of calves. Equation (4) explains how calf mortality was added as an additional cost to each remaining calf.

(Value of Newborn Calf + (Cost up to death × ((Interest Rate/365) × 60)) × Mortality Rate)/Remaining Calves(4)

Health costs are reflective of a standard vaccination protocol including fly control, respiratory vaccine, vitamin A, D, and E, selenium, and a vaccine for rotavirus and coronavirus scours, and *E. Coli*. Labor costs related to health tasks were compiled into a “working heifer” labor expense. The total health cost was figured at $9.22 per calf. Fair market prices were assumed on all vaccine and health-related equipment through averaging online prices obtained in January 2019.

The prevalence of respiratory illness and diarrhea was determined by the 2014 Heifer Raiser Survey conducted by the USDA, 18% for respiratory illness and 25% for diarrhea on average. Because of the variation in this measure from farm to farm it was made stochastic to account for variation between farms. The minimum incidence was 16% for respiratory illness with a maximum of 19%; the minimum of diarrhea was 22% with a maximum of 28%. Based on the selected prevalence, there was a direct relationship to the additional treatment cost for each calf. We modeled a protocol that would include electrolytes and 3 days of antibiotics. We assumed an 85.6% improvement rate and culled the remaining heifers at the end of that week.

### 2.5. Statistical Simulation

A simulation model was developed in Excel 2013 (Microsoft, Redmond, WA, USA) utilizing @RISK and PrecisionTree add-ons (Palisade Corporation, Ithaca, NY, USA) to evaluate the cost of raising an individual heifer from birth to weaning under different management styles and systems. 10,000 simulations of the model were performed for each of the situations. Stochastic simulations allowed for variation of inputs values which are reflected in ranges of potential outcomes, unlike a static model which will always produce the same outcome. Modeling variables stochastically, such as weaning age, mortality rates, and disease prevalence, we can simulate different outcomes. All variables were modeled following a Pert distribution set with minimum, most likely, and maximum value. Assumptions were made based on published literature, surveys, and market assumptions were also used to calculate the total cost (Table 1). A month in the cost spreadsheet was considered 30 days. This model is available online at https://afs.ca.uky.edu/dairy/decision-tools, wherein all variables and assumptions can be modified to reflect different situations and individual farms.

## 3. Results and Discussion

### 3.1. Housing

The total cost to house calves in individual housing outside, individual housing inside and group housing were $21.12, $70.52, $94.30, respectively. All of these costs were within 1 SD of the average found in published literature. For housing that included a barn, the barn payment per heifer was the largest contributor to cost, while bedding was the largest contributing cost per calf for individual housing outside (Table 3).

### 3.2. Feed

Feed cost was heavily dependent upon the amount of milk allotted per day. Table 4 shows the total cost of each milk source with 6, 8, 10, and 12 L allotments. As milk allotment per calf increased, the cost of milk increased.

The cost of pasteurizing whole milk ranged from 10 to 18% of the total cost of feeding calves in applicable scenarios. This model assumed the same nutritional value and gain from milk replacer and whole milk, creating a limitation in the model. However, calves fed pasteurized or unpasteurized whole milk have been shown to increase model-produced ADG by at least 0.03 kg/day with the potential to be over 0.25 kg/day of gain in comparison to milk replacer [15]. The additional cost to feed whole milk has the potential to be offset by an increase in weight gain.

The estimated cost per kg of gain decreased as milk allowance increased, and with increasing ADG, shown in Table 5. For example, group-housed calves on milk replacer with an automatic feeder fed 6 L will cost $3.50 per kg of gain. When these same calves are increased to 12 L the cost decreases to $2.67 per kg of gain. The minimum decrease in cost was from feeding 10 L of milk replacer to 12 L of milk replacer at $0.01 difference per kg of gain, and the maximum savings per kg of gain was $0.41 increasing from 10 to 12 L of pasteurized whole milk. If birth weights were 44 kg with a goal of weaning calves at 100 kg, we could assume a minimum of $0.56 to $22.96 in feed efficiency savings per calf alone. Modeling cost per kg of gain following experimental data presented in the (NRC, 2001) equations indicates that feeding calves a higher allowance of milk decreases the cost per kg of gain. The cost of milk and calf starter, with our current assumptions in inputs and ADG, decrease cost per kg of gain.

### 3.3. Labor

The labor decisions depended on the housing system selected. Hourly wages for management are higher than those for paid employees as shown in Table 1. Employees contributed more to the total cost than management in conventional and automatic systems even though their hourly rate is lower. Labor costs associated with the automatic calf feeder were responsible for 23% of the total labor cost. Labor cost of individual housing and group housing contributed 33% and 26%, respectively. The minutes and total cost per hourly laborer were decreased from inside individual housing to group housing by 36% per calf for a value of 2.4 minutes or $0.50 per calf per day. This shows a reduction in overall labor cost but an increased demand for fixed and variable expenses. These include the paying for the feeder, annual maintenance and a barn to house calves.

This breakdown of cost follows the same trends of Wisconsin surveys of conventional and automatic calf raisers. The paid labor cost alone was reduced by 39% for farms utilizing an automatic calf feeder, and paid management decreased by 14%. The total pre-weaning cost decreased 6% from conventional to automatic labor; the cost difference was recovered in an additional fixed variable cost of the automatic calf feeders.

### 3.4. Health

Mortality rate and prevalence of diarrhea or respiratory illness, which were included in variable costs, impacted the total cost. The average cost, including the risk of each calf being healthy or experiencing diarrhea, totaled (mean ± SD) $5.39 ± 14.42 per calf. The average cost per BRD case was $0.70 ± 7.33 per calf. Preventative health costs added an additional $9.22 to each calf. 

The change in total cost per calf, accounting for additional expenses with fewer calves as mortality rate increases (2%, 8%, 10%, and 15%), are reported in Table 6. As mortality rate increased, the cost of infrastructure and higher cost management systems showed a larger increase in the dollar amount added for each calf. Across management styles, decreasing the mortality rate from 15% to 2% reduced overall cost from $39.47 to $36.84 per calf. For a farm raising 500 pre-weaned calves annually, potential savings by decreasing mortality 10% alone could be over $18,000.

It has been found that management practices specific to a housing type can change illness prevalence. For example, calves housed in groups of 12–18 had a higher incidence of respiratory illness and lower daily gains than calves housed in groups of 6–9 [9]. We assume a constant square footage per calf, therefore the barn square footage increases as the number of calves increase and this may not always be reflective of true management practices. A limitation to the model is the same probabilities in averages and ranges in mortality for all management pathways for calculated cost. 

### 3.5. Total Cost of Management Scenarios

All possible combinations of management decisions (each combination of housing type, milk source, and labor type) and for each of the 4 milk allotments were analyzed for total cost, daily cost, and percentage of feed, labor, and fixed and variable costs (Table 7). Fixed costs included barn and housing infrastructure, depreciation of assets, and interest. Variable costs included health-related expenses, mortality, and utilities for electricity and water. Feed represented the largest factor in all management scenarios, followed by labor, then variable and fixed costs. This follows the same results found in previously published models where 57% of total cost were due to feed costs [6]. 

Using the assumptions in Table 1, on average, the most expensive management style was the one utilizing group housing, feeding pasteurized whole milk with conventional labor. The least expensive management pathway was the one utilizing individual housing outside, feeding whole milk with conventional labor. The main difference in cost can be attributed to the larger infrastructure needs for group housing and the additional cost of a pasteurizer. Total and daily cost for all management scenarios with 6, 8, 10, and 12 L allotments are shown in Table 3.

The mean for total cost ranged between $258.56 to $582.98 per calf across all management pathways. As seen in previous literature, the mean cost in each milk allotment has less variation than when looking at the range of projected costs per management scenario. This can be attributed to variation in health and mortality rates. Increasing the mortality rate and disease prevalence increased the cost for the remaining calves by spreading infrastructure costs, the loss of the calf and incurred expenses, and additional illness treatments over fewer calves. Variation in costs is not always related to efficiency on-farm but instead related to trade-offs in management styles.

The least expensive pathways were the 3 combinations for individual housing outside. In these scenarios, housing cost contributed 7–8% of the total cost compared to other management pathways utilizing more infrastructure, where housing accounted for 21–30% of the total cost. The addition of barns with individual housing inside and in group housing was the contribution of the additional 14–23% of housing cost.

When costs were broken down by day, assuming a 65-day weaning age, the cost ranged from $3.83 to $6.19 per calf per day. The average daily charge for a contract raiser from birth to weaning was $1.88/day [16]. Based on our calculated total cost for rearing pre-weaning calves, this would create a significant loss for the contract raiser. But in the Wisconsin heifer raising survey the cost per day of fixed and variable costs, which most closely matches our model, $2.05–$8.73 for minimum and maximum daily cost [8]. This simulation model can be compared to surveys to validate the results are reflective of on-farm total values. Finally, this model can be used to estimate other housing situations other than the ones presented in the current survey, and herd numbers could be used to estimate heifer costs for individual herds. In this survey, we chose the most common management practices to raise dairy heifers in North America, but there are many other options of housing and feeding dairy heifer calves that require further investigation. 

## 4. Conclusions

Raising calves from birth to weaning contributes to a major portion of the total heifer raising cost. Milk and calf starter contributed over half the cost to raise a calf from birth to weaning. Costs calculated by this model are based on currently available data; it is likely some of our assumptions will under or overestimate total and specific costs of calf raising practices across the US. More data are needed to improve accurate assumptions for farms. However, no model will be able to accurately describe all situations of calf rearing in various locations. Calculating pre-weaning cost for each individual farm is critical in making management decisions and remaining sustainable.

## Figures and Tables

**Table 1:** Model inputs were adapted from the published literature, the latest USDA reports, and heifer raising surveys.

Variable	Value	Source
Number of pre-weaned calves in 2 months	84	Based on rearing 500 heifers annually
Employee Labor (/h)	$14.00	Based on National Dairy Labor Survey, 2014
Management Labor (/h)	$22.00	Based on National Dairy Labor Survey, 2014
Interest Rate	7%	
Barn construction per M^2^ Frame	$10.00	Adkins, 2017
Barn construction per M^2^ Frame and Group Pens	$15.50	Adkins, 2017
Individual hutch	$300.00	Based on average market price
Value of newborn calf	$100.00	Based on USDA market reports
Whole milk value (cwt)	$15.00	Based on USDA, 2016
Milk replacer value (22.7 kg)	$65.00	Based on average market price
Calf Starter (mt)	$550.00	Based on average market price
Automatic calf feeder value	$15,000	Based on (Adkins, 2017)
Pasteurizer value	$10,000	Based on average market price
Diarrhea prevalence	21.4%	Urie, 2018
Respiratory illness prevalence	12.7%	Urie, 2018
Pre-weaning mortality rate	5%	NAHMS, 2011
Water cost per calf pre-weaning	$0.50	Based on water price Jan. 2019
Electrical cost per calf pre-weaning	$0.50	Based on electrical price Jan. 2019
Bedding per calf pre-weaning	$11.00	Heinrichs, 2013
Weaning Age	65 days	Adkins, 2017

**Table 2:** Birth weight and weaning weight were a result of milk allotted and calf starter intake per calf following experimental data from Rosenberger et al. 2017. Average daily gain (ADG) followed the equation presented in NRC 2001.

Milk Allotment (L/day)	Starter Intake (kg)	ADG (kg)	BW (kg)	WW (kg)
6	64	0.3	40	77.4
8	63.7	0.3–0.6	40	87.6
10	63.4	0.6–0.9	40	98.4
12	60.3	0.9–1.2	40	108.3

Birth weight (BW) was assumed at 40 kg, weaning weight (WW) was calculated based on ADG for 65 day weaning age.

**Table 3:** Percentage breakdown of hutch/barn infrastructure, bedding and, water and electric on total housing cost per housing management decision.

Housing System	Individual Housing Outside	Individual Housing Inside	Group Housing
Hutch or Barn *	32%	83%	87%
Bedding	52%	16%	12%
Water & Electric	2%	1%	1%

* includes interest and depreciation of infrastructure.

**Table 4:** Cost of milk replacer, whole milk, and pasteurized whole milk as a milk source for calves with 6, 8, 10, and 12 L milk allowances.

Milk Source	Milk Allotment (L)			
	6	8	10	12
Milk Replacer	$81.52	$107.02	$132.53	$158.04
Whole Milk	$81.41	$108.38	$135.36	$162.33
Pasteurized Whole Milk	$99.79	$126.76	$153.73	$180.71

**Table 5:** Feed cost per kg of gain of pre-weaned calves fed milk replacer, pasteurized whole milk and whole milk.

Milk Source	Milk Allotment (L)			
	6	8	10	12
ADG (kg)	0.3	0.3–0.6	0.6–0.9	0.9–1.2
Milk Replacer	$3.50	$2.75	$2.68	$2.67
Pasteurized Whole Milk	$3.60	$3.45	$3.31	$2.90
Whole Milk	$2.98	$2.96	$2.92	$2.60

**Table 6:** Total cost under each management pathway per calf when mortality rate is set at 2, 8, 10, and 15%.

Management Pathway	Mortality Rate			
	2%	8%	10%	15%
Individual Housing Outside				
Milk Replacer-Conventional	$283.03	$298.74	$304.44	$319.87
Pasteurized Whole Milk-Conventional	$291.27	$307.26	$313.06	$328.75
Whole Milk-Conventional	$287.98	$303.85	$309.61	$325.20
Individual Housing Inside				
Milk Replacer-Conventional	$303.03	$319.40	$325.34	$341.42
Pasteurized Whole Milk-Conventional	$311.28	$327.92	$333.96	$350.30
Whole Milk-Conventional	$307.98	$324.51	$330.51	$346.75
Group Housing				
Milk Replacer-Conventional	$312.06	$328.73	$334.78	$351.15
Pasteurized Whole Milk- Conventional	$320.31	$337.24	$343.39	$360.03
Whole Milk-Conventional	$317.01	$333.84	$339.95	$356.48
Milk Replacer-Automatic	$293.10	$309.15	$314.97	$330.72
Pasteurized Whole Milk-Automatic	$301.35	$317.66	$323.58	$339.60
Whole Milk-Automatic	$298.05	$314.25	$320.14	$336.05

**Table 7:** Total cost mean, SD, min and max of each management pathway under 6, 8, 10, and 12 L milk allowances.

Management Scenario	Milk Allotment (L)															
	6				8				10				12			
	Mean	SD	Min	Max	Mean	SD	Min	Max	Mean	SD	Min	Max	Mean	SD	Min	Max
Individual Hutches Outside																
Milk Replacer-Conventional	$276.03	$16.77	$259.92	$407.56	$301.71	$16.82	$285.07	$433.73	$327.39	$16.87	$310.22	$459.90	$353.07	$16.93	$335.37	$486.08
Pasteurized Whole Milk-Conventional	$295.55	$16.81	$279.04	$427.45	$323.38	$16.86	$306.30	$455.82	$351.22	$16.92	$333.56	$484.19	$379.06	$16.99	$360.82	$512.57
Whole Milk-Conventional	$274.63	$16.77	$258.56	$406.13	$302.47	$16.82	$285.82	$434.51	$330.30	$16.88	$313.08	$462.88	$358.14	$16.94	$340.34	$491.25
Individual Housing Inside																
Milk Replacer-Conventional	$301.11	$16.82	$284.49	$433.12	$326.79	$16.87	$309.63	$459.29	$352.47	$16.93	$334.78	$485.47	$378.15	$16.98	$359.93	$511.64
Pasteurized Whole Milk-Conventional	$320.63	$16.86	$303.60	$453.01	$348.46	$16.92	$330.86	$481.39	$376.30	$16.98	$358.12	$509.76	$404.13	$17.04	$385.39	$538.13
Whole Milk-Conventional	$299.71	$16.81	$283.12	$431.70	$327.55	$16.87	$310.38	$460.07	$355.38	$16.93	$337.64	$488.44	$383.22	$16.99	$364.90	$516.81
Group Housing																
Milk Replacer-Conventional	$345.11	$16.91	$327.58	$477.97	$370.79	$16.97	$352.73	$504.14	$396.47	$17.03	$377.88	$530.32	$422.15	$17.09	$403.03	$556.49
Pasteurized Whole Milk-Conventional	$364.63	$19.95	$346.70	$497.87	$392.47	$17.02	$373.96	$526.24	$420.30	$17.08	$401.22	$554.61	$448.14	$17.15	$428.48	$582.98
Whole Milk-Conventional	$343.72	$19.91	$326.21	$476.55	$371.55	$16.97	$353.48	$504.92	$399.39	$17.03	$380.74	$533.29	$427.22	$17.10	$408.00	$561.66
Milk Replacer-Automatic	$339.97	$16.90	$322.54	$472.73	$365.65	$16.95	$347.69	$498.90	$391.33	$17.01	$372.84	$525.07	$417.01	$17.08	$397.99	$551.25
Pasteurized Whole Milk-Automatic	$359.49	$16.94	$341.66	$492.62	$387.32	$17.00	$368.92	$520.99	$415.16	$17.07	$396.18	$549.36	$442.99	$17.14	$423.44	$577.74
Whole Milk-Automatic	$338.57	$16.89	$321.18	$471.30	$366.41	$16.96	$348.44	$499.68	$394.24	$17.02	$375.70	$528.05	$422.08	$17.09	$402.96	$556.42
